# Computer vision algorithms to help decision-making in cattle production

**DOI:** 10.1093/af/vfae028

**Published:** 2025-01-04

**Authors:** P Guarnido-Lopez, Y Pi, J Tao, E D M Mendes, L O Tedeschi

**Affiliations:** Department of Animal Science, Texas A&M University, College Station, TX 77843-2471, USA; Institute of Data Science, Texas A&M University, College Station, TX, USA; Institute of Data Science, Texas A&M University, College Station, TX, USA; School of Performance, Visualization and Fine Arts & Institute of Data Science, Texas A&M University, College Station, TX, USA; Department of Animal Science, Texas A&M University, College Station, TX 77843-2471, USA; Department of Animal Science, Texas A&M University, College Station, TX 77843-2471, USA

**Keywords:** cameras, cattle, computer vision, deep learning, neural networks

**Implications**

• Computer vision represents a valuable tool for helping cattle producers make decisions.

• Deep-learning algorithms, especially neural networks such as convolutional neural networks, conduct image classification, segmentation, object detection, and feature extraction.

• Computer vision helps to estimate intake, body weight and gain, body condition score, health status, and reproductive performance of cattle.

• The main goal for the future is to set up computer vision on-farm to execute real-time algorithms.

## Introduction

In recent years, integrating computer vision technologies into precision livestock farming (PLF) management systems have the potential to transform how cattle producers collect, monitor, analyze, and optimize animal production. Livestock production, particularly in cattle farming, encompassing beef and dairy under intensive and extensive production systems, faces numerous challenges ranging from optimizing feeding practices to detecting and managing diseases. Traditional monitoring and assessment methods often rely on manual labor and subjective evaluations, leading to inefficiencies and inaccuracies. Computer vision (CV) algorithms, leveraging the power of artificial intelligence (AI), machine learning (ML), and deep learning (DL) offer unprecedented capabilities in automating tasks, extracting meaningful insights, and improving animal management in different production systems and assisting in the decision-making processes such as 1) improving economic impact through solving inaccurate feed inefficiencies (Tedeschi, 2019), 2) decreasing productivity losses due to early disease´s detection in dairy cows (Miles, 2009), and 3) improving labor efficiency by automating tasks and providing real-time insights. Although CV is applicable to all livestock animals, most of the current development of algorithms for usage are conducted for swine production, given the availability of resources and the relatively straightforward CV application for recording indoor-housed animals.

**Figure 1.**
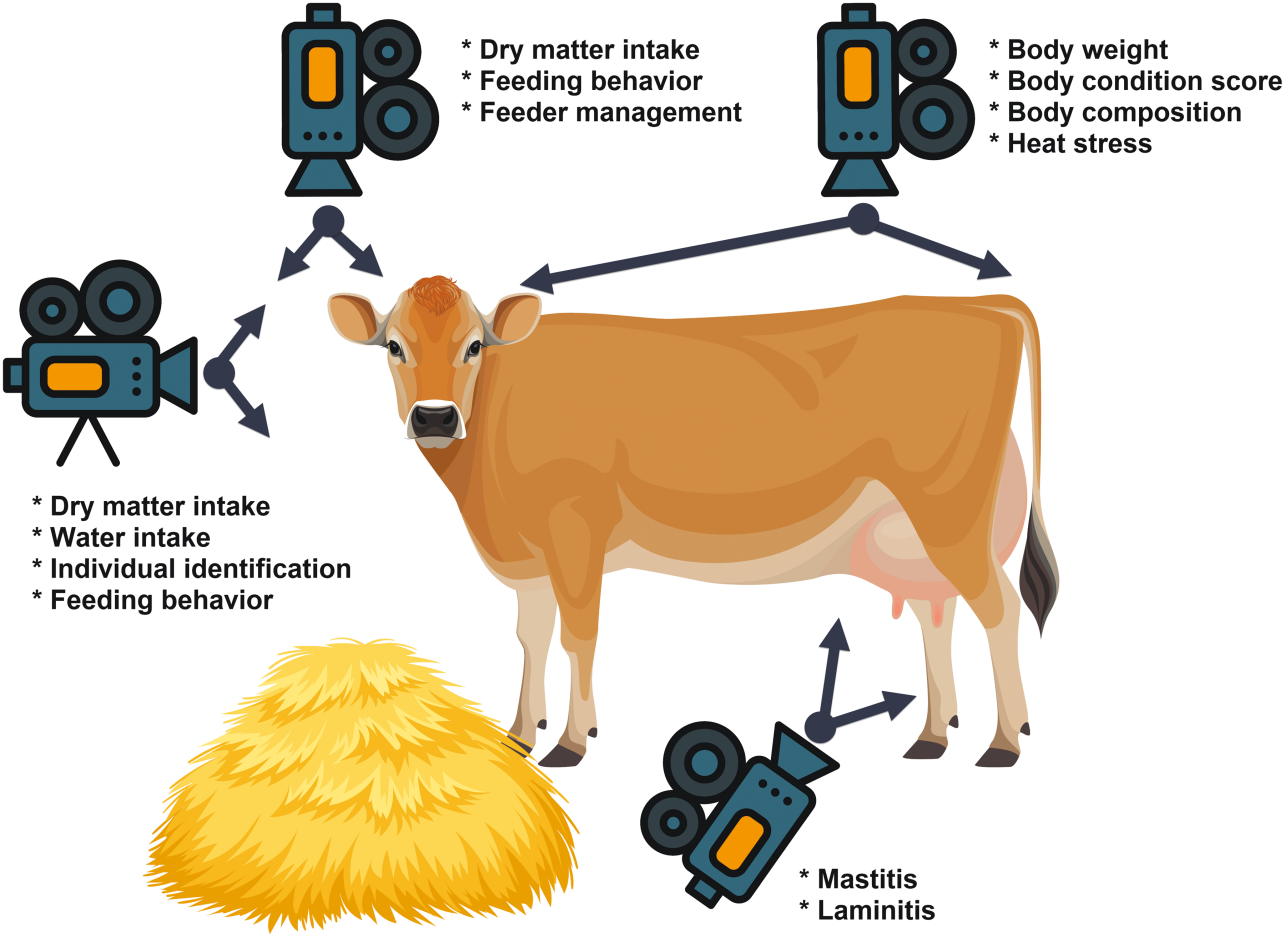
Schema that summarizes the placement of main cameras and the applications of the main computer vision algorithms in cattle prediction.

In this review, we aim to illustrate the use of cameras and the most utilized CV algorithms applied to cattle production, with a focus on their applicability in predicting key parameters such as individual feed consumption, feeding behavior, body weight (BW), body condition score (BSC), and the detection of prevalent cattle diseases including laminitis, mastitis, and heat stress (Figure 1). Furthermore, we discuss the advancements in cattle identification using CV techniques and their implications for enhancing the traceability and management of individual animals within the production system. By synthesizing the existing literature and highlighting the most applicable CV algorithms, we seek to provide insights into how these technologies can be applied on-farm to help decision-making processes for cattle producers and identify potential avenues for future research and technological innovation in PLF.

### Understanding deep-learning algorithms for computer vision

Computer vision algorithms are based on deep-learning neural networks, which are inspired by the functioning of the human brain through the connection of neurons to execute complex activities that require rationing (LeCun et al., 1988). The deep-learning architecture is composed of a sequence of multiple layers composed of neurons that transform an input into an outcome.

#### Basic structure and functioning of neural networks.

A general neural network is usually shown with lines representing the weights and circles indicating neurons, where each neuron is one value, just like every input and output neuron is one value. In the feed-forward pass, each neuron is calculated with equation (1) as follows:


$$\begin{array}{l} O^{k}=\sigma \left(\overset{n}{\underset{i}{\sum }}I_{i}^{k}w_{i}^{k}+b^{k}\right) \end{array}$$ (1)

where *O*^*k*^ represents the output of the neuron *k*; I is the previously connected neuron; w is the weight parameter, $b^{k}$ is the bias term for the neuron *k*, and *σ* is an activation function to ensure a positive output. In simpler terms, the formula describes how a neuron processes its inputs to produce an output. It takes the weighted sum of its inputs, adds a bias, and then applies an activation function to determine the final output. This process enables the neural network to learn and make predictions based on the input data.

One layer (vertical circles) can have many neurons and is fully connected with the previous input. The key to a successful neural network is the proper weights and biases in the model. The optimization of the collection of all weights $\vec{w}$ is called back-propagation, and the same applies to biases. A deep neuron network (hence the name DL) might have millions of weights and biases with hundreds of layers. These DL algorithms’ main goal is to minimize the estimated outcome’s prediction error through iterative back-propagation steps, which modify the weights of connections between neurons (Lee et al., 2018).

In each iteration, the difference between the prediction and the ground truth label (i.e., the observed values) measured using a loss function of f(prediction,label) called gradient descendent (∇). This gradient ∇ is a collection of partial derivatives concerning each weight (LeCun et al., 1988), written as $\left[\partial f\left(\vec{w}\;\right)/\partial w_{1}^{1},\partial f\left(\vec{w}\;\right)/\partial w_{2}^{1},\ldots \right]$. This function has the same dimension as $\vec{w}$ such that the new $\vec{w}$ can be updated using equation (2), as follows:


$$\begin{array}{l} \vec{w_{new}}=\vec{w}-\nabla f\left(\vec{w}\right)\times LR \end{array}$$ (2)

where LR is the learning rate, the ∇ is the gradient descendent function and $\vec{w}$ is the vector containing all parameters and/or variables (weights). In simpler terms, the formula describes how to update a model’s weights during training. First, calculate the gradient by computing the gradient of the loss function concerning the current weights; this gradient indicates how the loss function changes with small weight changes. Second, adjust the weights: subtract the product of the gradient and the learning rate from the current weights; this step moves the weights in the direction that reduces the loss function. This iterative process aims to find the optimal weights that minimize the loss function, thereby improving the model’s performance on the given task.

**Figure 2.**
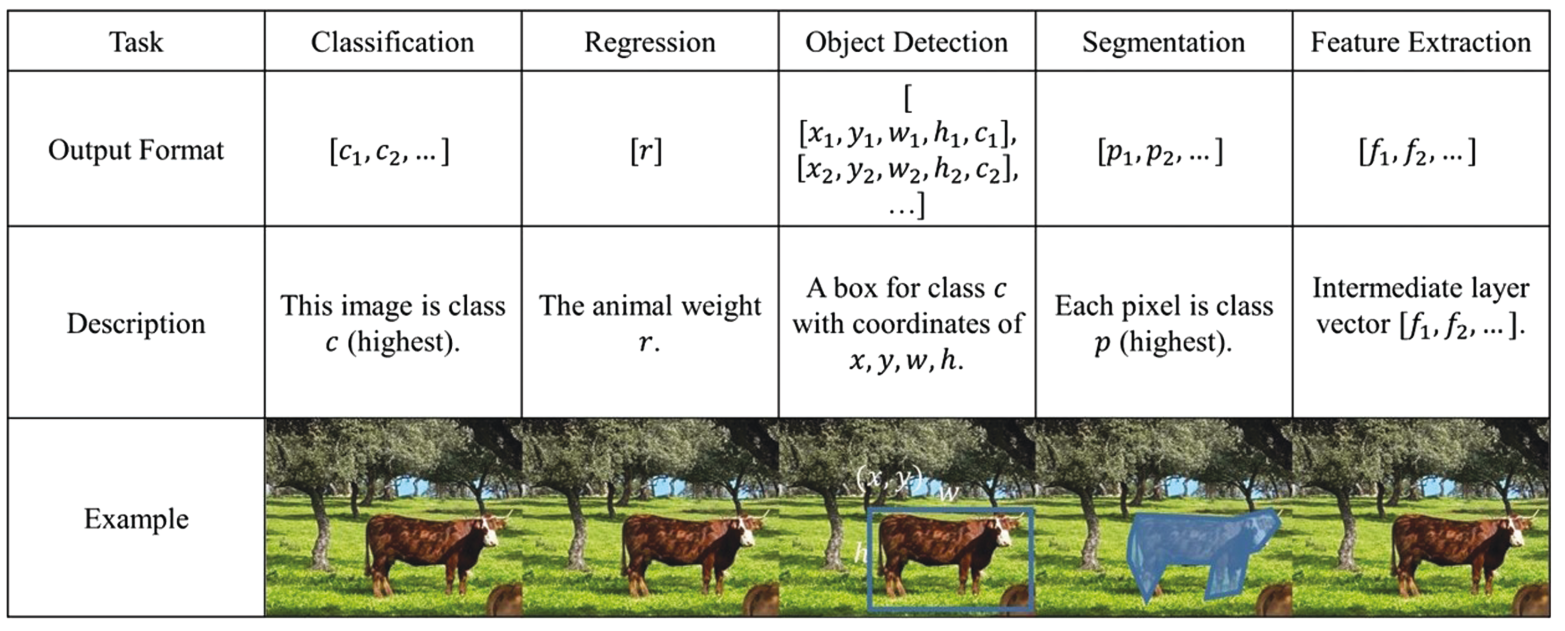
Example of different deep-learning algorithms used in computer vision over the same image.

In equation (2), the learning rate is a tiny number, such as 0.0001. After many iterations for training, these neural networks should be able to produce outputs close to the correct labels. Neural networks are highly flexible and can approximate any nonlinear relationships between known inputs and outputs. Given the nature of the CV input format, which is a matrix with 𝑤×ℎ×𝑐 dimensions, and the output format, which is typically one dimensional as probabilities and regression, the convolutional neural network (CNN) is the most successful so far because convolutional filters extract and preserve unique information. A general CNN model consists of different layers that compute convolved output, and it is also able to change image scale or size with max pooling. The fully connected layers produce the output the same way as the regular neural network mentioned above. Typically, thousands of different convolutional kernels (filters) are applied and optimized. The main functions of these DL algorithms in CV include (Figure 2).

#### Image classification and regression.

Each output neuron in a neural network represents either the probability that an image belongs to a specific class or a numerical value. The collection of these probabilities corresponds to the predefined classes (e.g., class1, class2). Typically, the class with the highest probability is selected. The loss function commonly used is cross-entropy, which measures the difference between the actual distribution of the labels and the predicted distribution output by the model. In the case of regression, the output neurons are the targets, and the loss function is a differentiable equation measuring the numerical differences, e.g., mean square error (MSE). This model structured for regression can be the same as classification, with convolutional (CONV) and max pooling layers, with the last output being numerical values such as age or BW. Successful classification/regression CNN models include ResNet, VGG series, and vision transformer. They established large classification datasets such as ImageNet or CIFAR.

#### Object detection.

It represents the categorization of the algorithm into region proposal- and regression-based methods. In the former approach, object regions are proposed for one or more categories in an image, whereas the regression-based method detects objects by framing their coordinates as a regression problem. One of the earliest prosperous region proposal-based techniques was the region-based CNN (RCNN). This algorithm generates a multitude of region proposals using selective search, followed by classification for each proposed box. The Algorithm you-only-look-once (YOLO) used anchor boxes to generate box proposals followed by a probability-based filtering process. Also, large object detection datasets include COCO and Objects365.

#### Image segmentation.

It consists of a CV technique that involves dividing an input image into meaningful regions or segments based on the semantic content of the image, and the primary goal of this algorithm is to classify each pixel in the image into a specific category or class. This image segmentation constitutes a foundational component of numerous CV frameworks, entailing the division of images into distinct segments or objects and assigning recognized classes to these segments. Some successful segmentation models include SegNet, U-Net, and PSPNet.

#### Feature extraction.

It consists of identifying and representing distinctive structures within an image. The intermediate features from the neural network middle layers are beneficial in categorizing objects and linking image content to pre-existing consolidated knowledge. For instance, classification and regression networks can discern recognized biological or structural features within images. Encoder–decoder networks excel in identifying the most compelling feature set describing a collection of images.

### How are deep-learning algorithms and cameras integrated into the computer vision structure?

Computer vision algorithms could be categorized based on their different input and output formats. The input for the CV system can be both 2-dimensional digital images (matrices) with multiple channels and videos. The most common types of images are red, green, and blue (RGB) colored images and extra channels, which include inferred or depth, which can provide shape and temperate information. However, images can only provide signals in a single instant. Videos, made of consecutive images (frames), can support more tasks because of the richer time-based information. On the output side, a typical task for CV is classification, which is a list of probabilities, each corresponding to a specific class. These probabilities can be mutually exclusive (one class per image) or multiclass classification (multiple classes for one image) for both images and videos, for example, lameness, feeding, and sickness. Other outputs in CV are the regression numbers such as BW and age, and others can be designed with one or several output neurons with the same intermediate neural network structures. Both classification and regression are well-studied in the CV community, and algorithms, including ResNet and vision transformer, can perform well in many tasks.

Moreover, combining both can detect objects with bounding boxes or pixel segmentations. In addition, beyond probability and regression, 3-dimensional point cloud reconstruction of animals can support detailed measurement and animal re-identification. Object tracking techniques are often integrated with object detection to identify individual animals.

Altogether, the categorization in Table 1 groups CV structures with similar input and output formats so they can potentially be re-used for transfer learning. Transfer learning, which is widely adopted in CV applications, is an effective way to deal with small datasets that are common in livestock research. The underlying principle is to pre-train a model on large datasets with millions of images, such as COCO or ImageNet. Next, most of the optimized weights and structures (backbones) will be slightly modified for specific tasks because it is easier and faster to transfer learning to a new model as the pre-trained model already learned the basic features of image recognition.

**Table 1.** Inputs and outputs of computer vision algorithms used in cattle with their respective functions

Input	Output	Applicable function in cattle	Research example
Image	Classification probabilities indicating different classes or situations	Classification of animal behavior (eating, resting, biting, chewing) and classification of health condition (e.g., lameness)	Rodríguez Alvarez et al. (2019)
Image	Regression of numerical values as a result of predictions (supervised learning)	Estimation of numerical values such as BW, dry matter intake, height, milk yield, or average daily gain	Gjergji et al. (2020)
Image	Segmentation probabilities for each pixel according to its corresponding class	Classification objects/items within images, such as collars, ear tags, health damages	Wu et al. (2020)
Image	Combinations of regression (e.g., bounding box coordinates) and probabilities (e.g., Mask RCNN with instance pixel masks within the bounding boxes)	A lightweight and high-precision detection model based on the YOLOv4 framework, named GG-YOLOv4, is used to automatically detect ocular surface temperatures from the thermal images of dairy cows	Wang et al. (2022)
Image or Video	3D reconstruction with point clouds	An unsupervised DBSCAN clustering algorithm was proposed to calibrate the leg region boundary based on clustering features	Li et al. (2022)
Video	Classification probability for sequential frames (e.g., drinking, estrus)	An algorithm for tracking the beef cattle’s key body parts, such as head–ear–neck position, using a state-of-the-art deep learning architecture, DeepLabCut. The extracted key points were analyzed using an extended short-term memory model to classify drinking and non-drinking periods	Islam et al. (2023)
Video	Combination of classification, regression, object tracking ID, and downstream analysis	A CNN model, which included a tensor of 4-channel matrices of data, each with 480 × 640 pixels. The model design was inspired by ResNet CNN, which achieved the best results in an ILSVRC classification and detection competition	Bezen et al. (2020)

Abbreviations: CNN, convolutional neural networks; RCNN, region-based convolutional neural networks; YOLO, you only look once.

### Current utilization of main computer vision algorithms in cattle production

In modern cattle farming, the integration of CV algorithms is becoming increasingly prevalent. These algorithms offer innovative solutions to assist efficiency, productivity, and animal welfare within the precision livestock philosophy (Tedeschi et al., 2021). By harnessing the power of advanced image processing techniques and machine learning (ML) algorithms, CV systems can automate various tasks, ranging from individual animal monitoring to herd management.

### Predicting individual dry matter intake and feeding behavior

Various systems for measuring feed intake have been developed, such as electronic scales installed in feeding stalls to track the feed consumed by individual cows. Multiple researchers have utilized these weighing mechanisms (Chapinal et al., 2007). However, both custom-designed weighing systems and commercially available options are predominantly accessible to research institutions rather than commercial dairy farms. This limitation is attributed to their high costs, additional infrastructure requirements, elevated maintenance needs, and the necessity for frequent cleaning, rendering them impractical for most commercial operations.

**Figure 3.**
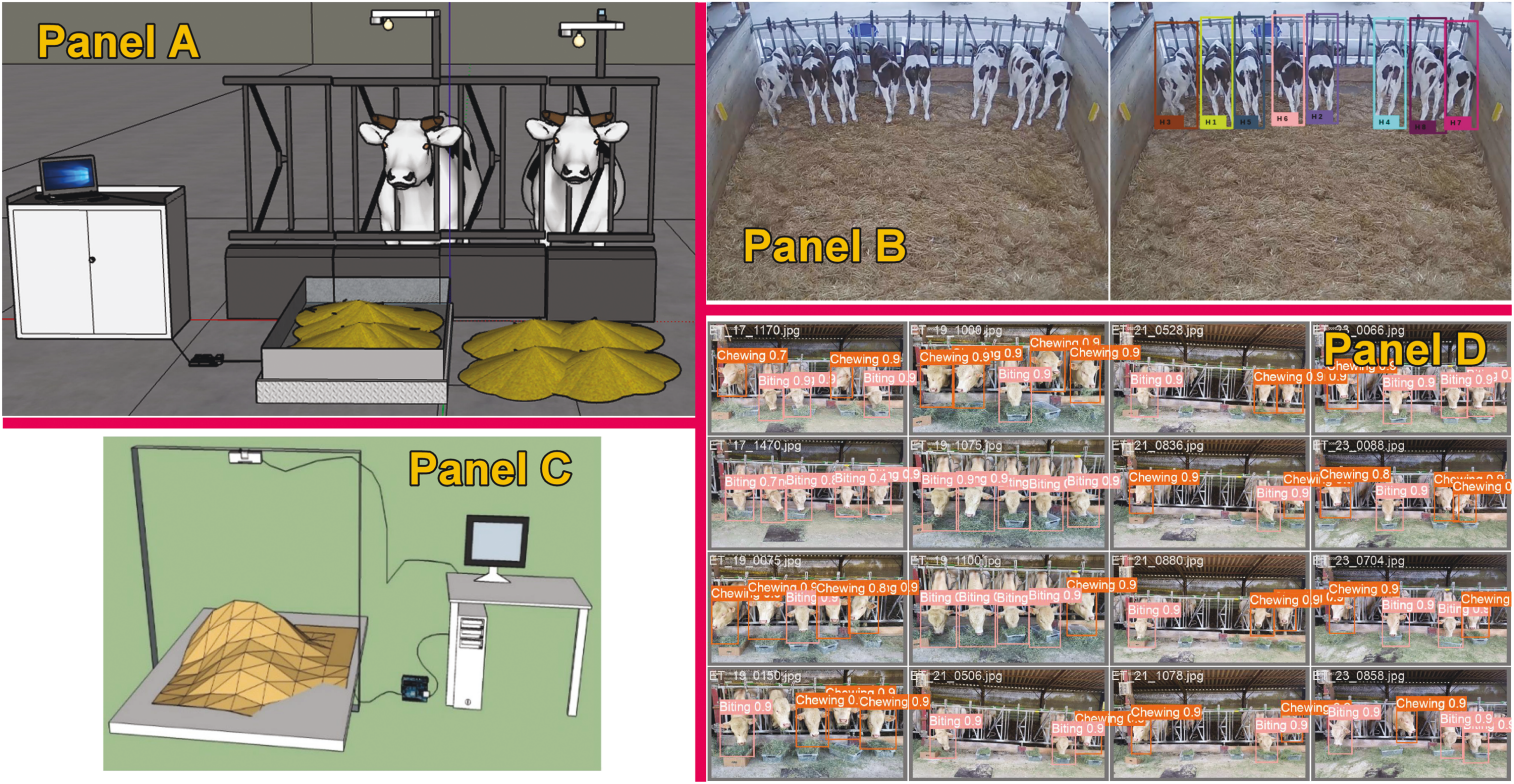
Example of studies using computer vision in cattle to predict individual feed intake. Panels A (Saar et al., 2022) and B (Bezen et al., 2020) predict individual intake through the estimation of feed mass in the feeder, while panels C (Bresolin et al., 2023) and D (Guarnido-Lopez et al., 2023) address intake through the estimation of feeding behavior or feeding time.

Therefore, CV algorithms arrived with an enormous possibility of helping to record on-farm individual intake in cattle. For this, 2 different methods exist the calculation of the feed mass or volume in the feeder and the determination of feed intake from the recorded feeding time. An image processing algorithm can be employed to assess the weight of the feed. Evaluations of feed mass utilizing cameras have been conducted through various methods, including structured light illumination techniques, light detection and ranging sensing methods (Shelley et al., 2016), and the utilization of 3D time-of-flight cameras under shaded conditions to mitigate interference from infrared light present in sunlight. DL, particularly CNN, constitutes a domain within the ML realm, adept at handling intricate machine vision tasks such as classification, detection, and recognition (Bezen et al., 2020). Few studies (Figure 3) have been conducted in the field of predicting individual intake from the feed mass determination by using CNN coupled to RGB-D cameras, showing promising results; Bezen et al. (2020) showed mean absolute error (MAE) and MSE 0.127 kg, and 0.34, respectively, in the daily feed intake of dairy cows, Saar et al. (2022) utilized a 2 transfer learning model based on CNN, showing an MAE of 0.12 and 0.13 kg per meal with a root of mean standard error (RMSE) of 0.18 and 0.17 kg per meal for the 2 different feeds evaluated in that trial in a range of 0 to 6 kg. Wang et al. (2023) used a Siamese network constructed to implement non-contact measurement of feed intake for dairy cows by training with collected data, showing an MAE and an RMSE of 0.100 and 0.128 kg in the range of 0 to 8.2 kg, respectively.

On the other hand, other authors decided to estimate individual DMI through the determination of feeding time by predicting feeding behavior through RGB-D cameras; Bresolin et al. (2023) showed accuracy on the feeding time prediction representing an *R*^2^ of 0.39, 0.78, 0.48, and 0.99 and an RMSE 0.78, 0.63, and 0.31 min for the number of visits, mean visit duration, mean interval between visits, and feeding time, respectively, using the YOLO v3 algorithm. Guarnido Lopez et al. (2023) used the YOLO v8 to determine individual feeding activities (biting, chewing, and visiting), showing an average high precision for all activities of 0.62, 0.83, and 0.81 in precision, recall, and average precision, respectively. After estimating feeding activities, the prediction of ingestion time (*r* = 0.82, *P* < 0.001) and, thus, the individual DMI (*R*² = 0.45, *P* = 0.001, RMSE = 0.18 kg DMI) resulted in highly promising. However, when using this method, it should be considered the possible concatenation of all prediction errors (error of the feeding activities recorded, error of the ingestion time from feeding activities, and error of DMI from predicted ingestion time) which may significantly decrease the accuracy of the DMI estimation. In addition, some other relevant factors that strongly impact the accuracy of these algorithms have to be considered, such as inter-object occlusion, camera position, or lighting conditions (Hu et al., 2021).

### Predicting individual BW and gain

**Figure 4.**
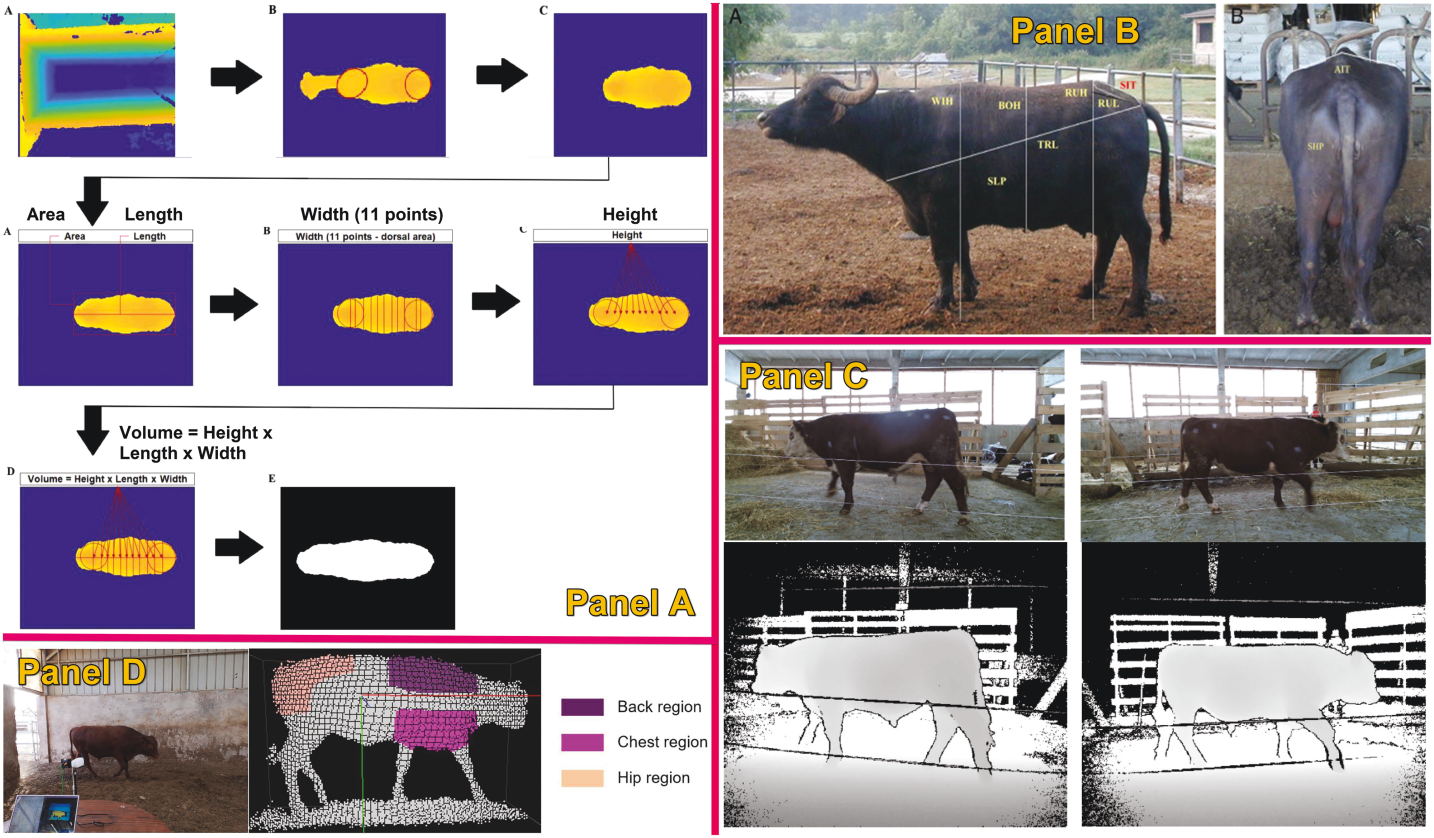
Example of studies using computer vision in cattle to compare body weight or average daily gain. Studies are shown in panels A (Cominotte et al., 2020), B (Negretti et al., 2008), C (Ruchay et al., 2022), and D (Hou et al., 2023).

Computer vision algorithms have also been proposed as a method for estimating both individual BW and cattle weight gain. In this case, most of the work conducted focused on extracting animal biometric measurements from RGB-D cameras or 3D images (Gomes et al., 2016) for the forecast BW from these biometric measurements (Gomes et al., 2016). The most utilized ML regression algorithms to predict BW from body measurements are linear regression, support vector regression adapted to the classification technique, the K-neighbors regressor, gradient boosting regression, the random forest regressor, and the most recent ones; multi-layer perceptron regressor, the light gradient boosting machine, and the extreme gradient boost regressor. Other authors have utilized one or some of these algorithms with RGB-D cameras in both top and side views (Figure 4). Core et al. (2008) estimated body length, HW, HH, surface area, and volume and then predicted BW from digital images, showing an *R*^2^ of 0.74, 0.79, 0.80, 0.50, 0.61, and 0.55, respectively. Similarly, Negretti et al. (2008) determined BW from the lateral area profile of animals and the profile area of hindquarters, showing an *R*^2^ of 0.94 and 0.92, respectively. Then, they established a multiple equation; BW = 427.7445 + 0.0431 × (lateral area profile) + 0.1263 × (hindquarters profile; *R*^2^ = 0.96, *P* < 0.001. Gomes et al. (2016) estimated BW from digital images taken through a Microsoft Kinect device, showing an *R*^2^ of 0.69, *P* < 0.001. In order to make this task more applicable, Gjergji et al. (2020) analyzed multiple CV algorithms (CNN, RNN/CNN networks, recurrent attention models, and recurrent attention models with CNN) to predict BW (average BW = 392 kg), being the CNN the one showing the highest performance in BW prediction (MAE = 23.19, RMSE = 38.46). Ruchay et al. (2022) utilized similar CV algorithms and cameras 2D cameras to the previous work but from a side view, achieving better BW predictions (*R*^2^ = 0.916). Also, Cominotte et al. (2020) evaluated the accuracy of the prediction of BW through 2D images using artificial neural networks in Nellore beef cattle, comparing the results across the different stages of growth, showing an RMSE = 8.6 kg and *R*^2^ = 0.91 for the weaning (average BW = 202 kg), an RMSE = 11.4 kg and *R*^2^ = 0.79 for Stocker phase (average BW=214 kg) and RMSE = 7.7 kg and *R*^2^ = 0.92 for the beginning of feedlot (average BW = 334 kg). More recent works directly tried to predict BW from the images without including body measurements. Sant’Ana et al. (2021) evaluated several machine-learning regression algorithms to predict BW from sheep images, obtaining great results (*R*^2^ = 0.687, MAE = 3.099) after applying a random forest repressor. Also, Xiong et al. (2023) utilized these regression algorithms to estimate BW with predicted cow’s volume from projected images, showing an *R*^2^ of 0.83 and an MAE of 19.2. More recently, other authors used 3D DL models to predict BW. Gebreyesus et al. (2023) indicated reasonable prediction accuracies with a mean correlation coefficient (*r*) as high as 0.94 in dairy cattle, while Hou et al. (2023) predicted BW with mean absolute percentage error (MAPE) of 3.2% through the DL algorithm PointNet++ in beef cattle.

Even when the CV algorithms have demonstrated their capability of successfully predicting BW of cattle, there are still some limitations that must be overcome to be able to evaluate the true potential of these techniques, including 1) relatively small numbers of animals per study, 2) different species and breeds, 3) inconsistent use of result measures and metrics across studies (e.g., RMSE, MAE, *r*, *R*^2^, accuracy, and correlation coefficients), 4) different experimental settings, 5) different 2D and 3D sensors, 6) various calibration approaches, and 7) the factors impacting technology acceptance by producers.

### Predicting body composition and BCS

**Figure 5.**
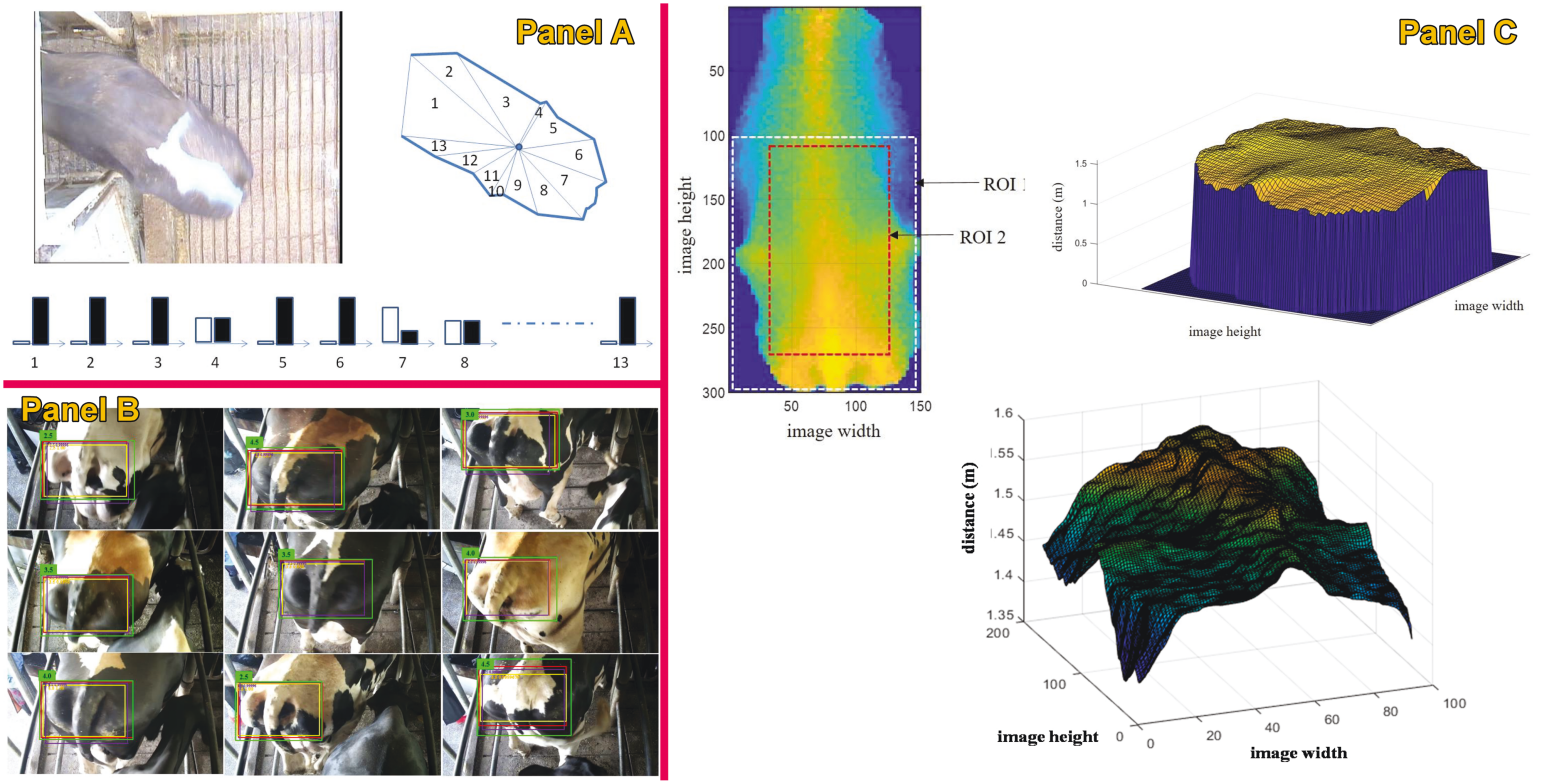
Example of studies using computer vision to estimate body condition score in cattle. Studies are shown in panels A (Battiato et al., 2010), B (Huang et al., 2019), and C (Zin et al., 2020).

Conventionally, BCS is typically acquired manually by a seasoned farmer through tactile or visual means. Nevertheless, this manual approach necessitates skilled farmers and is time-intensive. Moreover, the outcomes are subjective, susceptible to external environmental influences, and dependent on the assessor’s experience. Hence, there is a pressing need for objective, precise, and reliable BCS measurements. In the current livestock sector, sensors based on 2D and 3D technology are extensively employed (Figure 5) to gather information on cattle body parameters for BCS assessment. Vision, as a non-invasive method, is commonly utilized, typically involving 2 stages. Firstly, relevant visual features such as curvature, distance, or body contour are extracted. Subsequently, a regression model is constructed using these gathered features, either through manual construction or computer programming (Qiao et al., 2021). In addition, BCS prediction is highly dependent on the sensors utilized, mainly classified as 2D and 3D sensors. Regarding 2D sensors, Bewley et al. (2008) manually pinpointed anatomical landmarks on the top images and utilized angle features derived from these points to ascertain BCS, achieving an accuracy of 92.79%, Battiato et al. (2010) employed statistical shape analysis and regression machines to assess BCS, yielding a mean BCS error of 0.31, Huang et al. (2019) introduced the Sing Shot multi-box Detector method to detect the tail and evaluate BCS. Experimental results demonstrated that the enhanced Sing Shot multi-box detector method achieved a BCS classification accuracy of 98.46% and a location accuracy of 89.63% across 898 cow images.

However, more recently, 3D sensors have emerged as a promising technology for BCS assessment, offering richer body surface information compared to 2D or thermal image-based systems (Spoliansky et al., 2016). Currently, one of the prominent sensors for acquiring 3D data is the Time of Flight camera. The ToF cameras utilize visible or near-infrared light, with smart pixel sensors capturing the reflected light and measuring its return time. In Krukowski’s (2009) study, cows were manually photographed using a ToF camera, and features extracted from the dorsal and posterior regions demonstrated 100% accuracy in predicting BCS within a 0.5-point deviation from the actual BCS (considered favorable to have a 0.5-point variation between the ground truth score and the estimated score). Rodríguez Alvarez et al. (2019) utilized a Kinect v2 camera to capture top-view images of cows as they walked beneath it voluntarily, then applied the SqueezeNet model to estimate BCS, achieving an overall accuracy of 97% within a 0.50-point deviation from actual BCS. Zin et al. (2020) extracted 3D surface roughness parameters for BCS prediction using regression analysis, achieving a MAPE of 3.9% and a MAE of 0.13. Similarly, Stephansen et al. (2023) used the contour and back height features from 3D images as BCS predictors, together with class predictors (evaluator, herd, evaluation round, parity, lactation week), achieving > 93.5% of the coefficient of determination.

Despite the advancements in 2D sensor systems and methodologies mentioned earlier, it is important to acknowledge that 2D vision provides only a 2-dimensional animal projection. The absence of the third dimension in vision constrains applications that rely on depth information (Spoliansky et al., 2016). Therefore, advancements in BCS systems utilizing 2D/3D sensors and associated methodologies represent notable progress.

### Predicting health status

**Figure 6.**
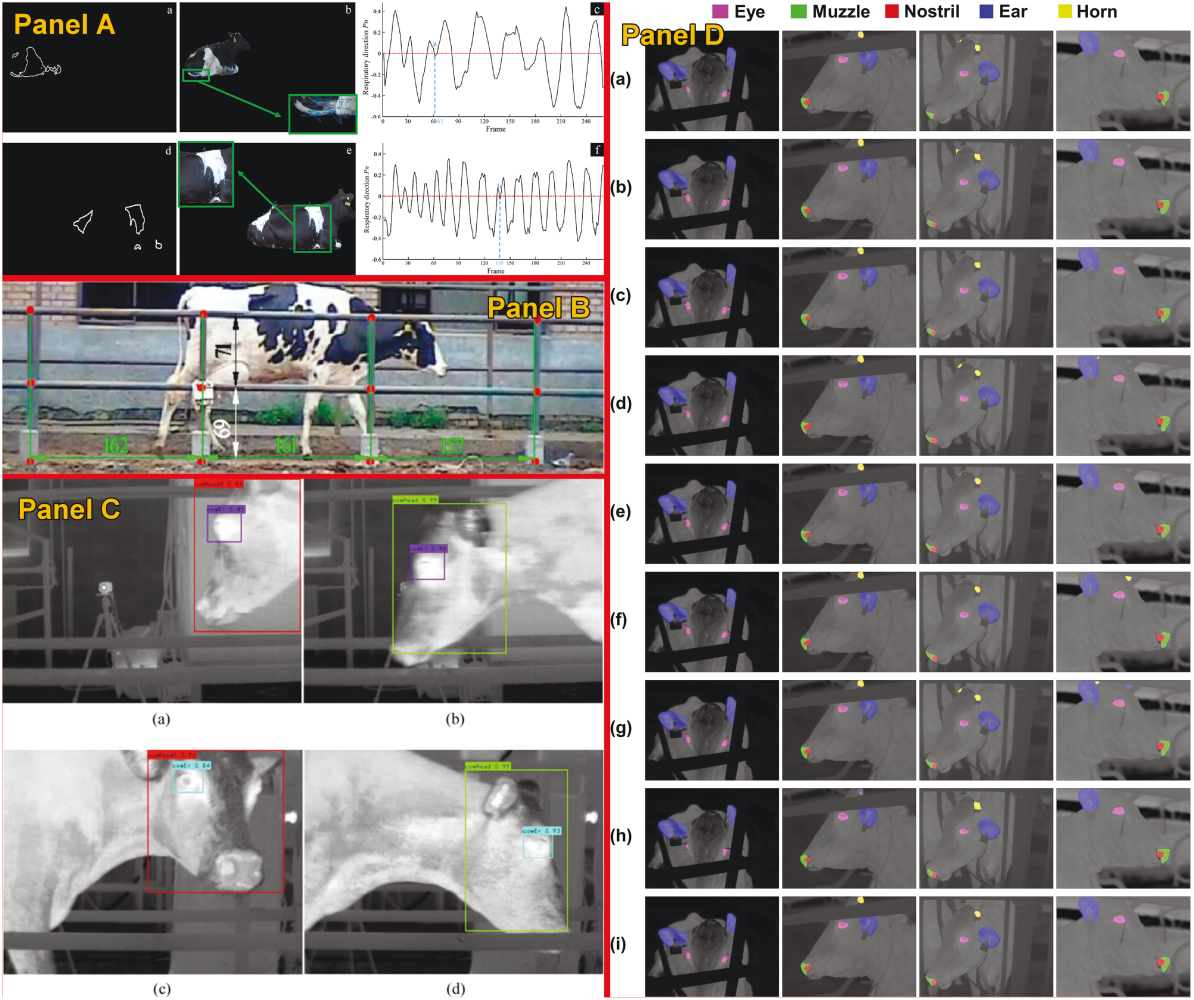
Example of studies using computer vision to estimate/predict health status in cattle. Studies shown in panels A (Wu et al., 2020), B (Zhao et al., 2018), C (Wang et al., 2022), and D (Shu et al., 2024).

Health status is a complex term encompassing all health parameters impacting animals’ health, such as diseases (bovine respiratory diseases, laminitis, and mastitis) or heat stress (Figure 6). Regarding bovine diseases, we will start with one of the most influencing in cattle production, the bovine respiratory disease (Miles, 2009). Respiratory patterns during resting time indicate a cow’s health status. For this, Song et al. (2019) employed the Lucas-Kanade method, which uses sparse optical flow to monitor the respiratory behavior of lying cows, and utilized it to detect movement in the abdominal speckle boundaries for respiratory behavior monitoring. Other authors utilized video magnification algorithms that enabled cow target segmentation and amplification of weak respiratory movements, achieving an accuracy of 93.04% (Wu et al., 2020). Wu et al. (2020) used a modified version of the YOLO algorithm (YOLACT) to identify cows and determine abdominal movements, achieving an average accuracy of 93.6% and an MAE of 3.42. Another relevant pathology in cattle production is laminitis, which has been studied through the evaluation of videos (Zhao et al., 2018). This is detected by evaluating changes in leg posture, where the template matching method was adopted to track the cow’s body and determine the cow’s leg positions; then, the 3-frame difference method was used to identify the moving cow’s legs (Zhao et al., 2018). Based on this characteristic, the researchers proposed an algorithm that considered this time and spatial differences using the local shape mutation caused by the static space of the cow’s hoof images as a feature for extracting the positions of the cow’s hooves.

Regarding mastitis, the leading health issue in dairy cows, Wang et al. (2022) used a method that combined the left and right udder skin surface temperature difference detection method with the ocular surface temperature through YOLO v5, achieving an accuracy, specificity, and sensitivity of mastitis detection of 87.62%, 84.62%, and 96.30%, respectively. Finally, addressing heat stress, Shu (2024) elaborated a thesis where they utilized the YOLOv5 to predict the temperature of several body parts, such as the vagina or eyes, and combined this information with respiration rate, cow variables, and environmental factors. This work predicted heat stress through respiration rate, which achieved a *r* = 0.95 and intra-class correlation of 0.98 compared with visual observation.

### Anticipating estrus and fertility performance

Other relevant parameters, such as reproductive traits, have also been evaluated through CV algorithms in cattle production. Regarding these reproductive traits, estrus detection to determine the best moment to inseminate cows has been identified as a factor highly impacting the economic profitability of cattle. Arago et al. (2020) utilized a tensor flow object detection algorithm with 2 custom CNN models trained to visualize cows’ reproductive behavior through bounding boxes, successfully identifying 50% of cows in heat.

## Challenges and Limitations

Even when CV algorithms have already demonstrated their capabilities in predicting useful variables to help make cattle production, some limitations still impact the accuracy of these models. One limitation is the lack of tested and trusted generalized models employed on new datasets. In CV applications, it is crucial to ensure that trained models can generalize to unfamiliar datasets or different animal species (Olubummo and Bello, 2024). However, due to the generalization gap in CV, testing a trained model on new datasets poses a significant challenge. This issue is particularly relevant in precision livestock farming based on CV (Kamilaris and Prenalefa-Boldu, 2018). Also, we can find some problems when the data that we want to estimate is non-linear, such as DMI or BCS. Regression problems cannot be described adequately using linear models, as many real-world problems are non-linear problems. In such scenarios, there are no suitable models better than non-linear models with non-linear functions for the description. Other problems are related to the algorithms by themselves, such as overfitting, representing whenever any techniques of ML that are capable of fitting known data that are meant for training ideally but cannot fit properly with external data, worsening the prediction accuracy (Mazo et al., 2024).

Besides these challenges, other problems related to cattle production should also be considered, such as the changes in environmental conditions. When setting up CV-based precision livestock farming, it is essential to consider environmental factors like climate, light, pollutants, water, food, population density, parasites, and sound, as they can negatively impact cattle farming and management. For instance, the inability to predict or control climatic conditions can result in unpredictable rain or drought. This consideration extends to the other mentioned environmental factors and socio-economic challenges, such as data ownership claims.

The prevailing lack of expertise in the cattle sector, particularly in emerging technologies, severely impedes the broader adoption of advanced tools such as computer vision (CV). In the realm of cattle farming, trained personnel are not just an asset but a necessity, as the effective management of such operations hinges significantly on human expertise. Moreover, the challenge of acquiring specialized knowledge and gaining relevant experience that is well suited to specific operational conditions compounds the difficulties faced in implementing CV-based precision livestock farming.

Additionally, the sector faces significant hurdles in converting academic and scientific research into viable, cost-effective solutions that can be practically applied in the field. However, a more pervasive issue remains the widespread misunderstanding and underestimation of mathematical modeling within the field of animal science. This misapprehension is particularly striking given the tremendous strides made in both large and small ruminant production over the last 60 years, driven by advances in mathematical modeling and data analysis (Tedeschi, 2019). This disconnect between theoretical advancements and practical application continues to be a significant barrier to the adoption of CV and related technologies in animal science.

## Future Directions and Opportunities

CV’s main future direction in livestock production is to help improve the efficiency and sustainability of animal production. Within these terms, we may consider CV as a valuable tool to collect/acquire real-time and on-farm data. Therefore, CV algorithms should be capable of collecting reliable on-farm data in real-time, which may be used to develop algorithms helping decisions of cattle farmers, such as the most efficient weaning weight of animals, applying nutrition precision techniques according to an accurate estimation of animal requirements (based on animal performance recorded through cameras), early detection of health status and diseases and a more accurate estrus determination to improve the fertility of the herd. For this, further research should be focused not only on improving the accuracy of these algorithms but also on conducting sensitivity analyses of predicted performance to determine to which extent CV algorithms could be applicable to farm conditions.

Additionally, it is essential to ensure that the next generation of researchers and practitioners is well-versed not only in AI and CV but also in the underlying biological mechanisms of animal production (Tedeschi, 2019). This dual focus will enable them to create more informed, practical CV applications that are deeply integrated with biological insights. Future research should, therefore, not only concentrate on refining the accuracy of these algorithms and conducting sensitivity analyses to evaluate the applicability of CV algorithms under various farm conditions but also on developing comprehensive educational programs. These programs should aim to equip students with a robust understanding of both the technological and biological aspects of animal science, ensuring a well-rounded approach to future innovations in livestock management.

## Conclusion

A variety of computer vision (CV) algorithms are employed to assess functional cattle performance metrics such as intake, BW, gain, BCS, health status, and reproductive traits. Among these, neural networks, particularly convolutional neural networks (CNN) and recurrent neural networks (RNN), have proven highly effective in accurately predicting these performance indicators. However, the deployment of this technology on farms faces significant challenges, including a lack of publicly available databases for validating and enhancing the algorithms and external factors like variable environmental conditions. Research aimed at addressing these issues is crucial. Looking ahead, these algorithms must evolve not only to predict animal performance accurately but also to do so in real-time and under actual on-farm conditions. This advancement will enable the use of such data to assist cattle farmers with decision-making, ultimately enhancing both the efficiency and sustainability of their operations. Thus, ongoing development and refinement of these technologies are essential to fully realize their potential in improving livestock management.

## About the authors

 **Dr. Guarnido-Lopez** is a postdoctoral research assistant in the Department of Animal Science at Texas A&M University. He completed his PhD in animal nutrition and physiology at INRA (France). In his first postdoc, he started working on computer vision in the University of Burgundy (France), trying to predict animal performance through RGB-D cameras. His research focuses on how to improve individual data collection of cattle individuals to determine performance and predict animal requirements, which will be afterwards used to develop precision nutrition (PN) algorithms. His main objective is to apply these PN algorithms on-farm conditions to help decision-making of cattle farmers by improving their feed efficiency and decreasing their environmental impact.

 **Jian Tao** is an assistant professor from the Section of Visual Computing & Computational Media in the School of Performance, Visualization & Fine Arts at Texas A&M University. He is also the Director of the Digital Twin Lab and the Assistant Director for Project Development at the Texas A&M Institute of Data Science. Tao received his PhD in Computational Astrophysics from Washington University in St. Louis in 2008. He is a faculty advisor of the Texas A&M Team for the SAE/GM AutoDrive Challenge Competition. Tao is an NVIDIA DLI University Ambassador and a contributor to the SPEC CPU 2017 benchmark suite. He is the Testbed Committee Co-Chair of the IEEE Public Safety Technology Initiative. His research interests include digital twin technologies, machine learning, numerical algorithms, data analytics, workflow management, remote sensing, information visualization, and high-performance computing.

 **Dr. Yalong Pi** is an associate research scientist at the Texas A&M Institute of Data Science (TAMIDS). With a diverse professional background, he previously worked as an Engineer at Gree Real Estate Co. Ltd in Zhuhai City, China from 2013 to 2016, and as a junior architect at Shanghai Water&Rock Group in 2017. He completed his post doc training at the Texas A&M Institute of Data Science. Prior to that, he obtained a Ph.D. in Architecture/Consrtuction from Texas A&M University (2020), an MS of Civil Engineering (2013) from Wuhan Institute of Technology, and a BS in Mechanical Engineering (2011) from Wuhan Textile University. He is a certified Architectural Engineer in China. Pi’s research focuses on computer vision applications in disaster reconnaissance, transportation information retrieval, precision livestock farming, and medical confocal imagery.

 **Mr. Mendes** is a graduate research assistant in the Department of Animal Science at Texas A&M University, currently pursuing his PhD in Animal Science. He holds a master’s in animal science from Texas A&M, obtained in 2010, and a DVM degree from the Veterinary School at UFMG (Federal University of Minas Gerais; Brazil), obtained in 2007. His employment history includes roles such as Technology Consultant at Prodap (DSM-Firmenich; Brazil), Data Scientist at GrowSafe Systems (Vytelle; Canada), TechAgr (Brazil), and and 8 years as a Research Support Analyst at EMBRAPA (Brazilian Agricultural Research Corporation). During his journey, he led and contributed to numerous projects in Brazil, the United States, Canada, and Mexico, closely aligned with precision livestock farming. Mr. Mendes’ research focuses on optimizing beef cattle production through advanced data-driven technology, leveraging analytics and data integration to enhance ranch productivity and sustainability.

 **Dr. Luis Tedeschi** is a professor, Texas A&M AgriLife Research Fellow, and Chancellor EDGES Fellow at the Department of Animal Science, Texas A&M University. He is also an Honorary Professor at the Dipartimento di Scienze Agraria, University of Sassari, Italy. Dr. Tedeschi holds a bachelor’s degree in Agronomy Engineering and a master’s degree in Animal and Forage Sciences from the University of São Paulo, Brazil, and a doctorate in Animal Science from Cornell University. His research interests focus on modeling the nutrition of ruminants, bioenergetics of growing cattle, and the development of decision support tools for the cattle industry. Dr. Tedeschi’s work involves integrating nutrition and modeling using in silico and in vivo research, combining cattle nutrition and management with artificial intelligence. He is recognized internationally for his accomplishments in predictive analytics of ruminant production. He has made significant contributions to the field through his innovative approach to energy partitioning in growing cattle. **Corresponding author:**luis.tedeschi@tamu.edu
